# 14–3-3 protein regulation of excitation–contraction coupling

**DOI:** 10.1007/s00424-021-02635-x

**Published:** 2021-11-25

**Authors:** Walter C. Thompson, Paul H. Goldspink

**Affiliations:** Department of Physiology and Biophysics (M/C 901) and Center for Cardiovascular Research, College of Medicine, University of Illinois at Chicago, 835 South Wolcott Avenue, RM E-202, Chicago, IL 60612 USA

**Keywords:** 14–3-3 proteins, Muscle, Excitation–contraction coupling

## Abstract

14–3-3 proteins (14–3-3 s) are a family of highly conserved proteins that regulate many cellular processes in eukaryotes by interacting with a diverse array of client proteins. The 14–3-3 proteins have been implicated in several disease states and previous reviews have condensed the literature with respect to their structure, function, and the regulation of different cellular processes. This review focuses on the growing body of literature exploring the important role 14–3-3 proteins appear to play in regulating the biochemical and biophysical events associated with excitation–contraction coupling (ECC) in muscle. It presents both a timely and unique analysis that seeks to unite studies emphasizing the identification and diversity of 14–3-3 protein function and client protein interactions, as modulators of muscle contraction. It also highlights ideas within these two well-established but intersecting fields that support further investigation with respect to the mechanistic actions of 14–3-3 proteins in the modulation of force generation in muscle.

## Introduction

Investigations into the function of 14–3-3 proteins over the last 30 years have elucidated their role in the regulation of several cellular pathways, including but not limited to, signal transduction, protein trafficking, cell cycle regulation, apoptosis, and metabolism. They operate as homodimers and heterodimers with each monomer capable of binding to phosphorylation sites within 14–3-3 binding motifs on client proteins. In general, there are two predominant binding motifs that have been identified, mode I and mode II each with consensus sequences defined as R(S/X)XpSXP and RXXXpSXP (X is any amino acid residue and p is adjacent to a phosphorylated serine or threonine) respectively [72]. A third consensus sequence (pS/pTX_1–2_-COOH) later identified appears to bind 14–3-3 proteins with a weaker affinity, although other binding motifs have been identified and the binding to unphosphorylated sites on some proteins is also recognized [15, 20, 29]. Once bound, they generally modulate client protein activation, inhibition, structural stabilization, masking of sites, and intracellular localization [47].

Phylogenetic analysis indicates 14–3-3 proteins evolved in unicellular and multicellular eukaryotes before the divergence of mammals. They exhibit a high degree of homology between the various isoforms of the same species, suggesting conservation of critical regions and function [53]. The high level of conservation of core regions and X-ray crystallographic studies of the mammalian isoforms has permitted identification of 3D structural features which are applicable to all the isoforms [73]. The ~ 30-kDa monomers consist of 9 antiparallel arranged α-helices (H1–9), forming an L-shape structure. Helices 3, 5, 7, and 9 form a highly conserved amphipathic grove on the inner surface of all the isoforms which forms the site of ligand binding on client proteins. The formation of homodimers and heterodimers occurs between highly conserved sequences in the N-terminus of helix 1 on one monomer, and helices 2 and 3 on the opposing monomer. The conserved nuclear export signal in helix 9 supports nuclear shuttling activities in addition to the established roles of the dimers acting as adaptors binding two different client proteins or two different regions of the same protein.

**Fig. 1:**
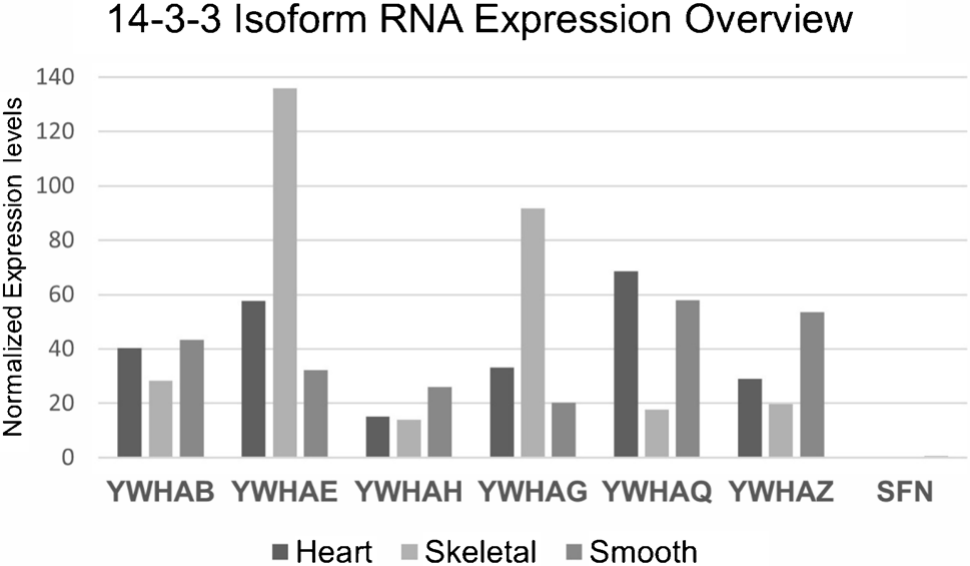
Human 14–3-3 isoform RNA expression in different muscle subtypes. Curated data from The Human Protein Atlas consensus dataset (www.proteinatlas.org). It represents expression levels from combined transcriptomic datasets that are normalized to 55 different tissues and 6 cell lines

In humans, the seven isoforms (β, ε, η, γ, θ, ζ, and σ) are encoded by separate genes (YWHAB, YWHAE, YWHAH, YWHAG, YWHAQ, YWHAZ, and SFN or Stratifin) and are expressed in a wide variety of tissues to varying degrees. Based on transcriptomic data in The Human Protein Atlas database, the 14–3-3 isoform-specific RNA expression profiles differ between the muscle subtypes and are summarized in Fig. 1. The data examining human 14–3-3 isoform-specific protein expression levels in the muscle subtypes are far less clear, with some isoforms not detectable. However, this may reflect limitations in the tools available to examine isoform-specific protein expression as opposed to the abundance of probe sets or sequence identification used in transcriptomic approaches. The regions of greatest protein sequence variability appear in the amino and carboxyl-terminal regions of the isoforms. The N-terminal region is responsible for dimerization and sequence variations here may underly the differing propensity of the isoforms to form homodimers or heterodimers [73]. Sequence variations in the C-terminus have been speculated to function in an isoform-specific autoinhibitory capacity during ligand binding, through a conformation change when phosphorylated [48]. Several excellent reviews have focused more extensively on 14–3-3 structure and function, their actions in modulating of cellular signaling, and the development of compounds to alter their activities, and are recommend for further reading [43, 47, 59, 71].

While 14–3-3 protein structure and function have been extensively studied in a vast array of non-muscle eucaryotic cells, the number of muscle-specific studies is limited. A recent PubMed search using the terms “14–3-3 AND muscle” identified 273 studies. Based on commonly used keywords listed, most of these studies examine 14–3-3 modulation of signaling pathways leading to transcriptional changes, apoptosis, autophagy, endoplasmic reticulum (ER) stress, cell cycle regulation, and mitochondrial function. In the case of smooth muscle, studies examining cell migration and cytoskeletal reorganization are also represented. On a broader scale, 14–3-3 proteins have been also implicated in organ development, muscle growth/hypertrophy, muscle differentiation, substrate uptake, and metabolism. However, the purpose of this review is to summarize emerging studies examining the interaction of 14–3-3 proteins with the proteins associated with excitation–contraction coupling (ECC) and in some cases, aligning their function with signaling events associated with its regulation. Moreover, from the prospective of a greater understanding of ECC and its regulation, there is little recognition of the role of 14–3-3 proteins in this process in general. Consequently, we aim to draw attention to this area through the assembly of a body of literature and discuss the potential mechanistic implications for 14–3-3 regulation of muscle contractile function.

## Modulation of 14–3-3 expression and genetic manipulation of function in muscle

Studies examining the regulation of 14–3-3 expression have predominantly characterized changes in their expression profile with respect to injury or disease, but surprisingly none has investigated the molecular regulation of 14–3-3 gene transcription per se. The proliferation of vascular smooth muscle cells (VSMC) in response to vascular injury contributes to vascular restenosis. In the carotid artery following balloon angioplasty in rats, 14–3-3γ RNA expression increases within 24 h in the vessels. In cultured VSMC following serum, proinflammatory cytokine, and mitogenic stimulation, increases in 14–3-3γ RNA expression occur within 2–3 h and protein increases occur within 8–16 h [6, 7]. A possible translational corollary associated with these observations is indicated in patients diagnosed with coronary artery vasculopathy which is associated with cytokine-induced activation and proliferation of medial VSMC as a critical cellular event in restenosis. Compared to patients with end-stage heart failure, coronary artery samples show elevated 14–3-3γ protein expression which colocalizes histologically with smooth muscle α-actin in the media and neointima of the vessel [5]. Although most isoforms are expressed at low levels in many tissues, 14–3-3γ appears to be expressed at higher levels in human brain, skeletal muscle, and heart compared to other tissues [6, 7]. Similarly, 14–3-3ε appears to be expressed at higher levels in the human heart and skeletal muscle compared to other tissues and its expression peaks during late stages of embryonic development through neonatal life in the rodent heart [41]. Oxidative stress associated with taurine deficiency and streptozotocin-induced diabetes appears to induce the expression of 14–3-3σ in isolated cardiac myocytes, which is otherwise undetectable, but may play a role in cell cycle arrest in concert with DNA damage and cell death [23, 24]. Myocardial injuries associated with severe burn and sepsis induce 14–3-3γ and η expression changes within the first 12 h post-injury but not 14–3-3ζ, which was not detected in the heart [28]. Interestingly, a study modeling age-associated heart failure in the senescence accelerated prone mouse (SAMP8), 14–3-3η protein expression in the heart trended lower than the normal aging control, and was associated with cardiac dysfunction, dysregulation of markers of ER, myocyte DNA damage, and expression of pro-inflammatory cytokines [57].

Neonatal rat cardiac myocytes (NRVM) are extensively used as an in vitro model to examine aspects of myocyte biology and the expression of 14–3-3 β, γ, ε, and ζ isoforms can be detected at both RNA and protein levels. The expression of 14–3-3 proteins does not appear to change in response to norepinephrine (NE)-induced hypertrophy. However, inhibition of their activity via adenoviral mediated delivery of a 14–3-3 inhibitor (R18, a 20-mer peptide) is sufficient to induce and potentiate NE-stimulated protein synthesis, suggesting 14–3-3 proteins may suppress hypertrophic signaling pathways [38]. Similarly, 14–3-3 protein expression increases over a 12–24-h time-course in NRVM exposed to anoxia preconditioning as an in vitro model of late-stage ischemic preconditioning in the heart, but the response of specific 14–3-3 isoforms here was not studied [12]. Likewise, in anoxia-reoxygenation injury in H9c2 cells often used as a “cardiac-like” cell line, knockdown of sirtuin (SIRT2), a signaling protein involved in stress-tolerance, results in increased 14–3-3ζ protein levels implicating an involvement in cytoprotective signaling [42].

Proteomics-based approaches have helped to further define 14–3-3 isoform expression in muscle tissues. 14–3-3γ protein expression in the corporal smooth muscle cells of the corpora cavernosa has been shown to increase within days following streptozotocin-induced diabetes [74]. Likewise, quantification of 14–3-3 protein isoform abundance identified 14–3-3ε, γ, η, θ, and ζ/σ in differentiated C2C12 cells and mouse triceps, with greater levels of 14–3-3γ, η, and θ in the skeletal tissues compared to the other isoforms [16]. Analysis of 14–3-3 protein expression changes through the incorporation of isobaric tags and mass spectrometry analysis used to examine the tibialis anterior muscle proteome following sciatic nerve transection identified increased 14–3-3 β, γ, ε, and ζ abundance during the first week of denervation [60]. Since most studies have focused on 14–3-3 protein function in the modulation of transcriptional programs, very few have focused on 14–3-3 genes as targets of transcriptional regulation in the context of muscle growth. Examining the regulatory role of microRNAs (miRNA) during skeletal muscle myogenesis, ectopic expression of miR-34b, and quantitative proteomic analysis confirmed the downregulation of 14–3-3γ occurred as a direct target of miR-34b suppression via binding to the 14–3-3γ mRNA 3′UTR [61]. Likewise, the transcriptional modulation of 14–3-3γ and other cell cycle checkpoint genes in proliferating skeletal myoblasts may be associated with silencing the activity of the transcription factor MEF2C, which is normally most active during the terminal differentiation stages in myogenesis [8].

One of the earliest studies testing 14–3-3 function in muscle expressed a dominant-negative form of 14–3-3η in the heart. Dominant‐negative 14–3-3 mutant forms were first identified in Drosophila [10]. Expression of a DN-14–3-3η transgene in cardiac myocytes demonstrated the necessity of 14–3-3 function in preserving cardiac function and survival in response to pressure overload. All DN-14–3-3η transgenic mice died within 7 days of transverse aortic constriction [70]. Follow-on studies utilizing this DN-14–3-3η model have subsequently demonstrated the role of 14–3-3η in mediating apoptotic signaling intermediaries in response to pressure overload, diabetes, ventricular remodeling, and oxidative stress, and collectively implicate that enhanced 14–3-3 expression or activity may provide a therapeutic benefit in several cardiac disease states [25, 26, 54, 62, 68, 75].

Studies employing germ-line deletion of the 14–3-3 isoforms were mainly developed for the purpose of studying neurological defects associated with each isoform. However, they have provided somewhat conflicting data with respect to the necessity 14–3-3 isoform expression for survival and ability to thrive, in large part due to the influence of background strain and knockout strategies used. Originally, deletion of 14–3-3ε allele resulted in heterozygous mice that were normal and fertile. Breeding these mice to homozygosity mainly produced mice that then die within hours of birth, but with only a small percent surviving to adulthood if bred on a mixed background compared to original inbreed strain [64]. Examination of possible causality of premature death during embryonic development of the 14–3-3ε-deficient mice on the inbred strain revealed that 14–3-3ε expression in the cardiac myocytes was required for normal ventricular morphogenesis and compaction of the myocardium. Interestingly, expression of the other 14–3-3 isoforms did not change or compensate for the lack of 14–3-3ε, indicating a level of isoform specificity in the developing heart [34]. In a follow-on and more detailed study of the cardiac malformations resulting from 14–3-3ε deletion, defects in the outflow tract, atrioventricular endocardial cushions, valvular, and vasculature were all apparent during mid and late gestion indicating the necessity of 14–3-3ε to control multiple aspects of cardiac development [22]. Similarly, deletion of 14–3-3θ/τ results in death during embryonic development of homozygous embryos with indications of delayed cardiac development, but with otherwise a normal morphologic appearance. Interestingly, haploinsufficient offspring are viable with normal cardiac function, but show an enhanced rate of mortality and increased cardiac myocyte apoptosis following coronary artery ligation [36]. Deletion of 14–3-3γ by insertion of the neomycin gene into exon 2 on an inbred background did not produce any obvious phenotypic alterations despite initial gene copy number and offspring being born with the expected Mendelian frequency [58]. However, deletion of 14–3-3γ using gene trapping to insert β-galactosidase into exon 2 to produce a truncated protein with just the 3 N-terminal α-helical domains intact produced homozygotes that all died by weaning [32]. Mice deficient in 14–3-3ζ also generated by gene trapping and maintained as heterozygous breeders on an inbred background or a mixed background appear viable and phenotypically indistinguishable to their wild-type littermates [11]. Thus, except for 14–3-3θ/τ, no other studies have been conducted to examine the impact on 14–3-3 isoform haploinsufficiency in response to a perturbation in the muscles of these models, and none has examined muscle-specific 14–3-3 deletion.

## 14–3-3 modulation of muscle membrane channels, exchangers, and pumps

The fundamental function of muscle tissue is to generate force which is initiated by the process of ECC. ECC is the progression of molecular events which start with changes in the resting membrane potential of the sarcolemma resulting in an action potential. The depolarization phase of the action potential generates a rise in the intracellular calcium which binds to a calcium sensor protein, permitting the interaction of myosin with actin and the generation force. This finely orchestrated multi-step process is regulated at various levels and differs between muscle subtypes. The changes in electrophysiological state of the sarcolemma through movement of different ions across the membrane facilitated by channels and pumps generally represent the first level of regulation. The function of 14–3-3 proteins in this aspect of muscle physiology is varied ranging from membrane channel chaperonin partners, aiding channel subunit assembly to modulators of channel and pump activities. In the context of 14–3-3 protein-mediated channel trafficking from the Golgi to the cell membrane, a description of the steps involved has been the focus of an excellent review [56], but the broader physiological implications, 14–3-3 protein interactions with these client proteins in the context ECC, are highlighted in the studies below.

Voltage-gated sodium channels are responsible for initiation and propagation of the action potential. These channels are composed of a transmembrane α-subunit which has four repeat domains and forms the pore (I to IV), plus accessory β-subunits. 14–3-3η interactions with the cytoplasmic region of the α-subunit interdomain I were first identified by a yeast 2-hybrid screen and validated biochemically [1]. Similar interactions with 14–3-3θ/τ and ζ were also identified, and 14–3-3η was shown colocalized with the Na^+^ channel to the intercalated disc in cardiac myocytes. Functionally, the presence of 14–3-3η did not change channel activation or current density but altered channel inactivation in a heterologous cell system, which required in part 14–3-3 protein dimerization. Further examination of 14–3-3 protein interactions with the Na^+^ channel has shown they may support the formation of α-subunit dimers harboring mutations linked to cardiac arrhythmias. Inhibiting the 14–3-3 interaction abolished the dominant negative effect on current amplitude of the mutant channels. Binding site analysis indicated that 14–3-3 binding did not play a direct role in channel dimerization but altered gating properties [14]. While a second 14–3-3 binding site was identified in the interdomain I cytoplasmic loop in addition to the previously reported site, which 14–3-3 isoforms and their dimerization state in bridging these sites to account for the altered gating properties of these channels have yet to be defined.

The inward rectifier K^+^ current carried by the Kir2.1 channel reestablishes and maintains the resting membrane potential in cardiac muscle cells. While the function of these channels’ contrasts with that of the Na^+^ channels, a pool of these two channels appears to be trafficked to the membrane. There they exist in multiprotein complexes and reciprocally modulate each other’s channel density. Inhibiting 14–3-3 protein interactions does not appear to impact individual channel densities but abolished reciprocal modulation of the complex without altering the formation of the complex in the membrane [66]. Interestingly, it is proposed that disruption of anterograde trafficking of the Na^+^ channel via 14–3-3 protein interaction inhibition may occur within the same region of the interdomain I cytoplasmic loop initially identified to contain PKA phosphorylation and ER retention motifs, and subsequently identified to contain 14–3-3 binding sites [40, 76]. A similar proximity and potentially functionally overlapping area of ER retention and 14–3-3 binding motifs has also been recognized in the pore-forming subunit of the voltage-gated calcium channel Cav2.2, predominantly expressed in presynaptic nerve terminals. Here it was shown to play a role in membrane trafficking of the pore-forming subunit independently of the auxiliary subunits, through 14–3-3θ/τ binding and masking the ER retention signal thereby allowing the channel to escape the ER [37]. The co-existence and interplay between ER retention motifs recognized by the COPI complex of the retrieval pathway of protein containing vesicles from the cis-Golgi membrane to the ER, and 14–3-3 binding motifs required to reach the cell surface, were studied in the trafficking and assembly of the metabolically sensitive ATP-sensitive K^+^ (K_ATP_) channel complex in the heart [4]. Utilizing knockout mice deficient in the pore forming subunit (Kir6.2), the sulfonylurea receptor subunit (SUR1), which contains ER retention signal, was shown to be retained intracellularly localized to the Golgi in the ventricular myocytes. β-adrenergic receptor agonists stimulated the translocation of SUR1-containing K_ATP_ channel, and increased SUR1, Kir6.2, and 14–3-3 phosphorylation, indicating 14–3-3 protein interactions control anterograde trafficking needed to overcome COPI-dependent retrieval signaling.

Activation of the delayed rectifier channels carries the K^+^ current and modifies the rate of membrane potential repolarization during the action potential. The *ether-a-go-go-*related gene (*HERG*) encodes the pore-forming subunit of the channel permitting the delayed rectifier K^+^ current *I*_Kr_, responsible for the rapid phase of depolarization. Binding of 14–3-3ε enhances voltage-dependent activation in the negative voltages ranges in CHO cells that could result in a faster current activation during cardiac action potentials. This functional effect occurs through direct 14–3-3 protein cross-bridging and stabilization of the PKA phosphorylated state of the channel, which appears to involve two sites (N- and C-terminal site) [30]. The pathophysiological significance of 14–3-3 protein modulation of HERG channel properties was highlighted in the analysis of naturally occurring C-terminal truncation mutations in heterozygous families with long-QT syndrome (LQTS) [13]. These channels are an assembly of four α-subunits each composed of cytoplasmic N- and C-terminal domains, and six transmembrane segments. C-terminal truncation mutated channels, in which the PKA phosphorylation, 14–3-3 binding sites, and ER signal are removed, can still bind 14–3-3ε and assemble as functional channels in the membrane. However, mutant channels do not respond to hyperpolarized membrane potentials and display dominant-negative behavior when co-expressed with wild-type channels in CHO cells. While not studied directly in cardiac myocytes, modeling of this HERG channel activity on the action potential duration in response to sympathetic stimulation indicated 14–3-3 protein interactions may be important in suppressing arrhythmias arising from premature ectopic beats in patients with LQTS due to C-terminal channel truncation mutations. Exploring HERG channel 14–3-3ε interactions in the context of β-adrenergic modulation indicated that the β_1_-adrenergic receptor competes with the HERG pore-forming subunit for 14–3-3ε both in the presence and absence of receptor stimulation. β_1_-adrenergic receptor competition for 14–3-3ε, and complex formation, appears to be directly occurring through PKA phosphorylation of putative 14–3-3 binding sites within the receptor and can be decreased by β-receptor antagonist treatment. Studying these interactions in heart samples, the β-receptor/14–3-3ε complex coeluted to a greater extent following stimulation [65]. In studying the functional consequences on the HERG channel, current modulation following 14–3-3ε recruitment to the PKA phosphorylation sites identified in the cytoplasmic domain of the β-receptor showed that phosphorylation-deficient mutants did not associate with 14–3-3ε which abolished β-receptor modulation of the HERG channel current. Finally, investigation of drug interactions with mutant HERG channels in LQTS using patient-specific human-induced pluripotent stem cell–derived cardiac myocytes (hiPSC-CMs) implicated that peroxisome proliferator-activated receptor-delta (PPARδ) agonists may exert indirect effects on mutant HERG channel activity by inducing 14–3-3ε expression changes, but no direct data in support of this role was investigated [19].

While the literature surrounding 14–3-3 protein interactions with membrane channels probably represents the most in-depth area of investigation with respect to their functional role in modulating ECC, studies examining 14–3-3 interactions with membrane pumps and exchangers which contribute to ion homeostasis in muscle cells also provide examples of their influence. The Na^+^/K^+^-ATPase, an electrogenic pump which creates a gradient of Na^+^ and K^+^ across the plasma membrane to establish a resting membrane potential, is of note. The Na^+^/K^+^-ATPase activity increases during cardiac ischemia and adrenergic stimulation via the phosphorylation of a 72 amino acid accessory protein called phospholemman, which regulates Na^+^ pump function. Phospholemman’s interaction with 14–3-3β is increased during ischemia and in isolated cardiac myocytes following direct PKA activation but is abrogated by expressing a mutant 14–3-3β (K49Q) [21]. Likewise, 14–3-3β binding to the Na^+^/H^+^ exchanger (NHE) which regulates intracellular pH and mediates increases in exchanger activity during ischemia/reperfusion injury through phosphorylation of NHE1 by p90RSK. Uncoupling the 14–3-3/NHE interaction via overexpression of dominant-negative RSK in the heart was found to be beneficial and limited cell death [45].

## Calcium homeostasis

The rise in cytosolic calcium levels is the trigger for contraction in all type of muscles. The management of the intracellular calcium concentration reflects the balance of calcium influx and release from intracellular stores, verses its reuptake into stores and extrusion out of the cytosol. 14–3-3 proteins have been identified to interact with some of the exchangers and pumps that play an integral role in the maintenance of calcium homeostasis.

The plasma membrane Na^+^/Ca^2+^ exchanger and Ca^2+^-ATPase pump both function to remove and transport Ca^2+^ ions from the cytosol into the extracellular space. While different isoforms exist, both are enriched in muscles and have been shown to be modulated by 14–3-3 proteins. In the framework of Na^+^/Ca^2+^ exchanger interactions, the NCX2 isoform which is predominantly expressed in the brain was identified to interact with 14–3-3ε and ζ which produced an inhibitory effect on NCX2 function. Extending these observations to the NCX1 and NCX3 isoforms which are predominantly expressed in muscle, a similar inhibition of exchanger function was noted. Specifically, the binding of 14–3-3ε reduced the exchanger’s ability to clear stimulated increases in intracellular Ca^2+^, which occurred independently of changing NCX levels in the membrane and NCX phosphorylation [51]. Of the different plasma membrane Ca^2+^-ATPase pump (PMCA) isoforms that exist, the ubiquitously expressed PMCA1 and PMCA4 isoforms are expressed in muscle. Despite their wide tissue distribution, their functional properties are modulated in part by various partner proteins. These proteins interact with the different intracellular domains of the pump of which the N-terminal domain has the lowest sequence homology between the isoforms. Focusing of N-terminal domain interacting partners to assign PMCA isoform specificity, the binding of 14–3-3ε to this region in PMCA4 but not PMCA2 occurs independently of phosphorylation and inhibits the pump’s ability to export Ca^2+^ which is rescued by silencing 14–3-3ε gene expression. Interestingly, the N-terminal domain of PMCA4 does not appear to interact with 14–3-3θ and ζ isoforms based on their expression profile in HeLa cells possibly implying a level of 14–3-3 isoform target specificity, but the remaining isoforms were not tested [52]. Expanding the study of 14–3-3 interactions with PMCA1 and PMCA3, both pumps were found to interact with 14–3-3ε whereas PMCA3 also interacted with 14–3-3ζ. In this instance, testing all the other 14–3-3 isoforms against these two client proteins supported the notion that 14–3-3ε is specific for PMCA1 in HeLa cells. Functionally, both pumps were also found to inhibited by 14–3-3 interactions in the N-terminus [39]. Sequence analysis of the N-terminal region revealed that despite a conserved putative 14–3-3 binding motif present in all four PMCA isoforms, the predicted secondary structure of an adjacent α-helix in PMCA2 differed and may contribute to the destabilization of the 14–3-3 interaction noted with this isoform.

Probably the most compelling examination of 14–3-3 protein function in the context cardiac muscle ECC is the recognition that 14–3-3 proteins interact with Phospholamban (PLN) [46]. PLN associates with the sarcoplasmic reticulum Ca^2+^-ATPase pump (SERCA) to negatively regulate SERCA activity and limit the kinetics of Ca^2+^ uptake into the SR. Phosphorylation of serine 16 on PLN by PKA following β-adrenergic stimulation and threonine 17 by Ca^2+^-calmodulin-dependent kinase II removes the inhibitory effects of PLN on SERCA, increasing Ca^2+^ uptake and accelerating relaxation of the contractile apparatus. Leveraging prior appreciation of the interaction between Arg-Arg ER retention motifs recognized by the COPI complex and 14–3-3 binding motifs for assembly and trafficking of channels, the existence of these adjacent sequences in the N-terminus of PLN was investigated [46]. Through a series of biochemical approaches to test the existence of protein–protein interactions, PLN pentamers were captured using 14–3-3 protein pull-downs, proximity labeling experiments in neonatal cultured myocytes, and immunoprecipitation data supported evidence of a direct interaction. Moreover, the affinity of 14–3-3 to bind to PLN monomers, and the avidity of the monomers to form pentamers, was dependent on Ser 16 and Thr 17 phosphorylation. Physiologically, the 14–3-3 protein interaction with PLN was promoted following β-adrenergic stimulation and shown to slow the kinetics of PLN dephosphorylation by masking the phosphosite on PLN. Functionally, the time constant of the Ca^2+^ transient decay was shown to be prolonged following acute β-adrenergic stimulation in the presence of recombinant 14–3-3 protein, dialyzed into the adult myocytes. Thus, the 14–3-3 protein interactions with PLN serve to stabilize the phosphoform and disinhibit its effect on SERCA activity. Interestingly, the authors also point to a clinical corollary that may exist in patients carrying a mutation which results in the loss of Arg14, adjacent to the 14–3-3 binding site at Ser16. These patients develop an aggressive dilated cardiomyopathy and genetically engineered mice recapitulating this myopathy suffer premature death, highlighting a pathophysiologic consequence associated with the disruption of the 14–3-3/PLN interaction.

## The contractile apparatus

The contractile apparatus of muscle performs the fundamental role of generating force in response to a suitable stimulus. While elevated levels of intracellular calcium trigger the contractile response, differences exist in the calcium sensor which initiates the molecular events that culminate in crossbridge formation between the muscle subtypes. With the recognition of effector proteins 14–3-3 proteins interact with to modulate ECC, these studies point towards a more diverse role beyond the regulation of signaling pathways in muscle. However, only a few studies exist with respect to 14–3-3 proteins and the modulation of muscle contractile system. These studies mainly focus on 14–3-3 proteins in regulating upstream kinases and phosphatases that directly regulate contractile proteins to alter their function, or events associated with cytoskeletal rearrangement impacting contractile function, and are highlighted below.

In an effort to identify substrates of various protein kinases in skeletal muscle, phosphorylation of the skeletal myosin light chain kinase (MLCK) at Serine 16 was identified as a target for an upstream kinase [27]. MLCK phosphorylation of myosin light chain (MLC) plays a central role in the formation of the actin-myosin crossbridge in smooth muscle and modulates crossbridge function in cardiac and skeletal muscle. Phosphorylation of MLCK at Ser16 is associated with MLCK autophosphorylation and is increased during tetanic muscle contraction. Phosphorylation of Ser16 was shown to induce binding of 14–3-3 proteins but it does not appear to change MLCK activity [27]. However, while MLC phosphorylation is central to crossbridge formation in smooth muscle, equally important is MLC dephosphorylation by myosin light chain phosphatase (MLCP), to initiate muscle relaxation. MLCP is composed of 3 subunits, of which the myosin phosphatase targeting subunit (MYPT1) binds the enzyme to myosin. MYPT1 can be directly phosphorylated to alter its affinity for myosin and increase the phosphatase activity of the catalytic subunit, but also change its intracellular localization. In regulating these activities, biochemical studies have identified 14–3-3β to directly bind to MYPT1 to induce its dissociation from smooth muscle myosin, attenuate MLCP phosphatase activity, and alter its cellular localization [33]. 14–3-3β bound to the region surrounding Ser472 on MYPT1 results in increased MYPT1/14–3-3β binding upon MYPT1 phosphorylation and an associated increase in MLC phosphorylation, suggesting a mechanism by which contraction maybe sustained or augmented may exist due to the interaction of 14–3-3 proteins with MYPT1.

The control of smooth muscle tone and vascular diameter involves not only the balance of myosin-light chain phosphorylation and dephosphorylation, but additional mechanisms related to the dynamics of cytoskeletal reorganization. The cytoskeleton anchors the contractile apparatus to the extracellular matrix and contributes to the contractile response to vasoactive stimulation. 14–3-3 proteins interact with proteins involved in the regulation of actin filament dynamics, but their interactions with cofilin have gained significant attention with respect to the regulation of smooth muscle contractile function. Cofilin is an actin regulatory protein that binds to the side of F-actin to promote actin filament disassembly and recycling of actin monomers during cytoskeletal remodeling. Involved in mediating the cofilin/F-actin interaction is the small heat shock protein 20 (HSP20), also known as HSPB6. While the precise mechanism is still unclear, it is proposed that HSP20-mediated smooth muscle relaxation in response to elevated cyclic nucleotides results in HSP20 phosphorylation which displaces cofilin from its complex with 14–3-3 [17]. This hypothesis is based in part on the ability of a transducible recombinant phosphopeptide analog corresponding to 14–3-3 binding sequence surrounding Ser16 in HSP20, which has been shown to relax smooth muscle in various tissues, to disrupt the actin cytoskeleton, interact with 14–3-3 proteins, and dephosphorylate cofilin in cells. A comprehensive analysis of the 14–3-3 and HSP20 interaction showed that phosphopeptides derived from the N-terminus of HSP20 containing the site surrounding Ser16 interacted with and stabilized 14–3-3 proteins impeding their proteolysis [55]. The authors also explored the chaperoning activity of 14–3-3ζ monomers and dimers to prevent myosin S1 fragment aggregation subjected to increasing temperature. Compared to its homodimer and HSP20, the 14–3-3ζ monomer was the most effective in preventing S1 fragment aggregation, suggesting that 14–3-3 proteins may target myosin as a client protein to alter its properties. Extending this interaction, it has been shown that human 14–3-3σ directly interacts with the tail fragments of purified human non-muscle myosin IIA-C fusion proteins to alter their assembly kinetics independent of phosphorylation [69]. Examination of the direct interaction between the 14–3-3 isoforms and the non-muscle myosin isoforms revealed they all interacted to varying degrees, but collectively suggests that 14–3-3 proteins may have a direct role regulating cellular mechanics at the level of the contractile proteins. Intriguingly, modulating the molecular interaction between Hsp20 and 14–3-3 proteins has formed the basis of a high-throughput screen for the discovery of small molecule analogs of phosphorylated HSP20 that may provide a therapeutic regiment for the treatment of smooth muscle vasospasm in lung diseases [2].

In cardiac myocytes, the cytoskeleton plays an important role in maintaining and anchoring the sarcomere and contributes to the overall tension development. Cytoskeletal rearrangements involving HSP20/14–3-3 and cofilin/14–3-3 protein interactions influencing actin cytoskeletal dynamics may have implications in the pathogenesis of heart failure. Treatment of cardiomyocytes with the HSP20 phosphopeptide which displaces cofilin from 14–3-3 proteins promotes actin filament disassembly, increased shortening and relaxation rates and the time constant of the Ca^2+^ transient decay [49]. Interestingly patients with a Hsp20 mutation P20L, which forms part of the 14–3-3 protein binding site surrounding Ser16, develop a dilated cardiomyopathy. Examining the impact of the human Hsp20-P20L mutation, it was shown this mutation conferred a diminished physical interaction with 14–3-3 proteins and failed to compete for 14–3-3 binding to cofilin upon phosphorylation [67]. Consequently, this mechanism may be sufficient to influence contractile dynamics directly or indirectly via modulating the cytoskeletal reorganization that are mediated through changes in the state of F-actin polymerization.

## How 14–3-3 interactions combine to modulate ECC in health and disease

**Fig. 2:**
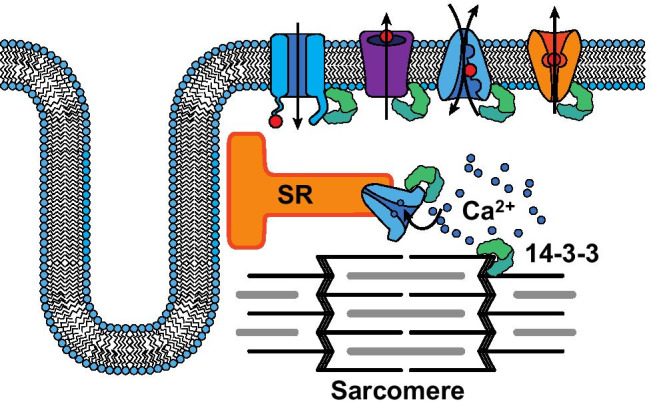
Summary of the main 14–3-3 and client protein interactions involved in the modulation of excitation–contraction coupling that have been identified to date. Specific aspects of client protein assembly into functional complexes are not depicted but are described in the text. (  14–3-3 dimer;  voltage-gated channel carrying inward current (e.g., Na^+^ channel);  voltage-gated channel carrying outward currents (e.g., K^+^ channel);  cation exchanger (e.g., Na^+^/Ca^2+^ exchanger);  electrogenic pump (e.g., Ca^2+^ ATPase);  sarco/endoplasmic reticulum Ca^2+^ ATPase)

Without a clear indication as to necessity of 14–3-3 proteins in the modulation for ECC through muscle-specific deletion, speculation based on the literature presented herein and in other electrically excitable tissues does suggest their dysregulation may contribute to disease state. 14–3-3 proteins have been implicated in neurodegenerative and neuropsychiatric diseases and are highly expressed in the brain, making up about 1% of the total soluble brain protein [9]. In the context of ECC, the literature at present points towards three main areas which are summarized in Fig. 2. These are the regulation of electrophysiologic properties, regulation of calcium homeostasis, and modulation of contractile function, but with fewer studies existing in support of the later. Their role in the protein–protein interactions associated with voltage-gated channel assembly, modulation of current properties in response to physiological stimulation and membrane trafficking in the formation of channel complexes, all point to their need in maintaining the normal electrophysiologic state of the muscle cell membrane. Simulations modeling the effect of 14–3-3 loss-of function on the action potential indicate their absence may be proarrhythmogenic but this has not been tested directly. However, their involvement in the assembly of mutant channel subunits implicated with inherited arrhythmias indicates they play an indirect role in disease development via the assembly of dysfunctional client proteins. These data and those showing their regulation of ionic gradients by modulating the activity of membrane pumps and exchangers all coalesce around the notion 14–3-3 proteins exert critical control over membrane excitability, which constitutes the primary triggering event in ECC.

Until recently, their role in maintaining intracellular calcium homeostasis was associated mainly through the analysis in heterologous systems. However, the discovery that 14–3-3 proteins interact directly with phospholamban to alter reuptake kinetics of Ca^2+^ into the sarcoplasmic reticulum within the cardiac myocyte represents the most direct mechanism to date and provides pivotal support to their role in ECC. PLN plays an important role in muscle in relaxation albeit to differing degrees between the muscle subtypes. Extending the 14–3-3/PLN interaction to the regulation of contractility places this mechanism at a critical nexus in ECC. Moreover, the suggestion that this mechanism may be causal in the development of dilated cardiomyopathy in patients that carry a mutation in the 14–3-3 binding motif in PLN may provide rationale for the development of 14–3-3 targeted therapeutic intervention using small molecular stabilizers such as fusicoccin-A or derivatives [31].

Finally, while the data implicating direct 14–3-3 protein interactions with the contractile machinery are evolving, a recent publication aimed at defining alpha-actinin interacting partners at the Z-disc of the sarcomere identified both 14–3-3ε and 14–3-3γ using a proximity-dependent biotinylation (BioID) approach in hiPSC-CM [35]. To our knowledge, this represents the first study that demonstrates a 14–3-3 protein contractile protein interaction, and broadly implicates their involvement in myofibrillar assembly and/or trafficking of accessory proteins to the myofibrils.

## Concluding remarks and future directions

There is generally a greater understanding as to the functional significance of 14–3-3/client protein interactions in certain cellular processes and disease states. Undoubtedly, their role in the formation of signaling complexes, intracellular protein translocation, and protein trafficking of more than 200 client partners has rightly positioned them as important intracellular modulators [50]. Despite this recognition, the precise mechanisms of their interactions and composition of 14–3-3 dimers are still being elucidated, but as recognized in this review, answering these questions could have a significant influence on our understanding of ECC regulation and provide further therapeutic options for dysregulation of this critical process. To that end, new proteomic-based tools are being developed to better define the 14–3-3 protein interactome within different cell types, to quantify changes in these interactions in response to changes in the physiological and pathophysiological environment and help identify potential 14–3-3 binding domains. All bear brief mention as they are most applicable and would be useful for investigating the extent of 14–3-3 protein–protein interactions in ECC.

14–3-3 capture and release is an approach by which client proteins are captured via 14–3-3 affinity purification and eluted with a phosphopeptide analog corresponding to the mode I 14–3-3 binding domain consensus sequence (ARAApSAPA), for identification by mass spectrometry. As noted, this methodology first identified the breadth of the 14–3-3 protein interactions [50]. Modifications of this approach by which differential capture of 14–3-3 protein clients through isotope incorporation of digested proteins have permitted quantification of 14–3-3 protein/client protein interactions in response to signal pathway activation [18]. Limitations however have been the use of the yeast 14–3-3 isoforms (BMH1 and BMH2) for affinity capture even though they correspond to mammalian orthologs, but they do not permit isoform-specific capture analysis. Also, the use of mode I 14–3-3 binding domain phophopeptide to elute proteins which may not compete for all 14–3-3/client protein interactions. Nevertheless, the repertoire of 14–3-3 client proteins defined to date has helped define their scope of function in several cellular processes and permitted the formation of databases and bioinformatic tools which can be leveraged to identify and better understand 14–3-3 protein function in ECC. Likewise, an exciting alternative is the application of tandem affinity purification (TAP), an epitope-tagging purification strategy to purify protein complexes followed by tandem mass spectrometry. Evolving this approach, a TAP transgenic mouse line that expresses 14–3-3ζ was developed and used to identify interacting proteins in various tissues in situ [3]. One of the advantages offered by isolating 14–3-3 isoform protein complexes from their physiological environment is the analysis of tissue-specific interactions that might not be otherwise present in heterologous cell cultures. Further adaptation of this methodology to drive cell-specific expression, and/or breeding onto a disease model background, could provide an integrated physiological analysis of 14–3-3 protein client protein interactions during disease progression. In addition to these methodological approaches, as mentioned bioinformatic and in silico prediction web-based resources provide access to a database and a prediction method, for identification of potential 14–3-3 binding motifs within client proteins, and are helpful resources to initiate investigation into targets of interest [44, 63]. Utilizing the web-based 14–3-3 Pred tool built as a prediction method to analyze and prioritize putative 14–3-3 binding sites in > 2000 potential interactor proteins, we analyzed the major human contractile and calcium handling proteins expressed in the heart. This tool confirms some of the pre-existing targets discussed herein, but also identifies relevant new targets that are yet to be explored in the context of 14–3-3 modulation of ECC (Table 1). Of note is the presence of some well-defined serine/threonine phosphorylation sites within the putative 14–3-3 binding motifs such as S23 and S24 in the N-terminus of cardiac troponin I which play a significant role in the myofilaments response to Ca^2+^.

**Table 1:** Identification of putative 14–3-3 binding sites in contractile and calcium handling proteins associated with ECC in human cardiac myocytes using the web-based 14–3-3 Pred tool (http://www.compbio.dundee.ac.uk/1433pred.). Protein name and UniProtKB ID (in brackets), plus all sites predicted (strong and weak scores) except where noted. Proteins absent from Table 1 due to no 14–3-3 predicted sites include as follows: TNNC1_Troponin C, slow skeletal and cardiac muscle (P63316), TNNT2_Troponin T, cardiac muscle (P45379), MYL2_Myosin regulatory light chain 2, ventricular/cardiac muscle isoform (P10916), MYL7_Myosin regulatory light chain 2, atrial isoform (Q01449), MYL3_Myosin light chain 3 (P08590), MYL4_Myosin light chain 4 (P12829)

Myofilament proteins	14–3-3-predicted sites	Ca^2+^ handling proteins	14–3-3-predicted sites
TNNI3_Troponin I, cardiac muscle (P19429)	S23, S24, T31, S77, T143, S150, S166, S210	AT2A2_Sarcoplasmic/endoplasmic reticulum calcium ATPase 2 (P16615)	S136, T172, S265, S493, S495, S509, T847, S941, S973, S1006
TPM1_Tropomyosin alpha-1 chain (P09493)	S247, S271	PPLA_Cardiac phospholamban (P26678)	S16, T17
MYPC3_Myosin-binding protein C, cardiac type (Q14896)	S242, S275, S284, S286, S304, T498, T 688, T729, T1026, S1040, S1141, T1153, T1184, S1231	RYR2_HUMAN Ryanodine receptor 2 (Q92736)	53 sites-highest scored: T279, T301, S742, T1466, S1662, S0231, S2808, S3196, S4260, S4539
MYH7_Myosin-7 (P12883)	S111, S158, T446, T547, T665, T786, S810, T971, S1362, S1366, S1478, S1596, S1843, S1924	NAC1_Sodium/calcium exchanger 1 (P32418)	S101, S285, S392, T621, T836, T912
ACTC_Actin alpha cardiac muscle 1 (P68032)	T91, S201, T262, S340	AT2B4_Plasma membrane calcium-transporting ATPase 4 (P23634)	T60, T137, T315, S576, S590, T696, S756, T1102
ACTN2_Alpha-actinin-2 (P35609)	T57, T165, S291, T495, T564, S596, S624, T799, S870	CAC1C_Voltage-dependent L-type calcium channel subunit alpha-1C (Q13936)	36 sites-highest scored: S465, T688, T1462, T1622, S1718, S1879, T1953, S1975, S1981, S2027, S2098

In the context of further studies of 14–3-3 protein function in the field of ECC, many questions remain to be addressed. First, how to identify the physiologically relevant receptors and their regulation by 14–3-3 protein modulation within the context of ECC regulation? This is a vital but understudied area despite strong data in heterologous systems showing 14–3-3 protein interactions with β-arrestin and its role in G-protein-coupled receptor signaling. Second, what role do 14–3-3 isoforms play in mediating different pathways and functional activity of downstream proteins in the pathogenesis of muscle diseases? Third, given the proclivity of 14–3-3 isoforms to form homodimers or heterodimers, but also function as monomers, does this enable them to fulfill specific roles in ECC? Fourth, what effects do 14–3-3 proteins exert on client proteins in the balance of phosphorylation and dephosphorylation of critical residues in the regulation of ECC? These may be challenging questions to purse in muscle due to the unique structure functional relationship of the multimeric protein assemblies involved in ECC, and the potential for multiple 14–3-3 interaction sites within a single protein. Nevertheless, one clear advantage to help tackle these questions related to the structural, temporal, and spatial regulation of 14–3-3/client protein interactions in the regulation by ECC is the well-defined physiological and biophysical readouts associated with each of aspect this process in muscle.

## Abbreviations

β-receptor: Beta adrenergic receptor
CHO: Chinese hamster ovary
COPI: Coat protein complex 1
DN-14–3-3: Dominant negative 14–3-3 protein
ECC: Excitation contraction coupling
ER: Endoplasmic reticulum
HeLa: Henrietta Lacks cervical cancer cells
HERG: Human ether-a-go-go gene
hiPSC-CM: Human-induced pluripotent stem cell–derived cardiac myocytes
HSP20: Heat shock protein 20
LQTS: Long QT syndrome
MEF2C: Myocytes-specific enhancer factor 2C
MLC: Myosin light chain
MLCK: Myosin light chain kinase
MLCP: Myosin light chain phosphatase
MYTP1: Myosin targeting phosphatase subunit 1
miRNA: MicroRNA
NCX: Sodium calcium exchanger
NE: Norepinephrine
NRVM: Neonatal rat ventricular myocytes
PKA: Protein kinase A
PLN: Phospholamban
PMCA: Plasma membrane calcium ATPase
SAMP8: Senescence-accelerated mouse-prone 8
SERCA: Sarcoplasmic reticulum calcium ATPase
SITR2: Sirtuin 2
TAP: Tandem affinity purification
VSMC: Vascular smooth muscle cells
3′UTR: 3 Prime untranslated region
P90RSK: P90 Ribosomal S6 kinase
